# Gene-Environment Interaction between the *IL1RN* Variants and Childhood Environmental Tobacco Smoke Exposure in Asthma Risk

**DOI:** 10.3390/ijerph17062036

**Published:** 2020-03-19

**Authors:** Yongzhao Shao, Yian Zhang, Mengling Liu, Maria-Elena Fernandez-Beros, Meng Qian, Joan Reibman

**Affiliations:** 1 Division of Biostatistics, Department of Population Health, School of Medicine (SOM), New York University, New York, NY 10016, USA; yian.zhang@nyulangone.org (Y.Z.); mengling.liu@nyulangone.org (M.L.); meng.qian@nyumc.org (M.Q.); 2 Department of Environmental Medicine, SOM, New York University, New York, NY 10016, USA; 3 Division of Pulmonary and Critical Care Medicine, Department of Medicine, SOM, New York University, New York, NY 10016, USA; MariaElena.Fernandez-Beros@nyulangone.org

**Keywords:** gene and environment interaction, environmental tobacco smoke, *IL1RN* variants, early onset asthma, SNP genotypes, risk haplotypes, case-control association study, population admixture, ancestral informative markers, inflammation

## Abstract

(1) Background: Variants of the interleukin-1 receptor antagonist (*IL1RN*) gene, encoding an anti-inflammatory cytokine, are associated with asthma. Asthma is a chronic inflammatory disease of the airway influenced by interactions between genetic variants and environmental factors. We discovered a gene–environment interaction (GEI) of *IL1RN* polymorphisms with childhood environmental tobacco smoke (ETS) exposure on asthma susceptibility in an urban adult population. (2) Methods: DNA samples from the NYU/Bellevue Asthma Registry were genotyped for tag SNPs in *IL1RN* in asthma cases and unrelated healthy controls. Logistic regressions were used to study the GEI between *IL1RN* variants and childhood ETS exposures on asthma and early onset asthma, respectively, adjusting for population admixture and other covariates. (3) Results: Whereas the rare genotypes of *IL1RN* SNPs (e.g., GG in SNP rs2234678) were associated with decreased risk for asthma among those without ETS exposure (odds ratio OR = 0.215, p = 0.021), they are associated with increased risk for early onset asthma among those with childhood ETS (OR = 4.467, p = 0.021). (4) Conclusions: We identified a GEI between polymorphisms of *IL1RN* and childhood ETS exposure in asthma. Analysis of GEI indicated that childhood ETS exposure disrupted the protective effect of some haplotypes/genotypes of *IL1RN* for asthma and turned them into high-risk polymorphisms for early onset asthma.

## 1. Introduction

Asthma is a chronic inflammatory lung disease with a large global burden [1,2,3]. A complex genetic disorder, asthma results from the interplay of multiple genetic variants with environmental factors [4,5,6,7]. Numerous genes associated with asthma susceptibility have been identified through candidate gene analyses and genome-wide linkage or association studies [7,8]. Recent studies highlight the importance of genetic variants of innate immune pathways [9,10,11] as well as gene–environment interactions (GEI) [4,7,12,13,14,15]. 

Members of the IL1 family participate in inflammation with well-established biological plausibility for an association between IL1 family genes and asthma or related phenotypes [16,17,18,19]. The IL1 signaling pathway involves the IL1 receptor type I (IL1R1), through which IL1α and IL1β induce a pro-inflammatory response. The interleukin-1 receptor antagonist (*IL1RN*) gene encodes the IL1 antagonist protein, IL1RA, which competitively binds to IL1R1 but does not elicit the downstream signal transduction cascade [18,19]. The pro-inflammatory effect of IL1 and the anti-inflammatory effect of IL1RA in asthma have been documented in human and animal functional and mechanistic studies [20,21,22,23,24,25,26,27]. Both IL1β and IL1RA are increased in bronchoalveolar lavage fluid during asthma exacerbations in humans [23]. IL1 is involved in the interaction between house dust mites and the innate immune response leading to allergic sensitization [28]. IL1RA also attenuates airway hyper-responsiveness following exposure to ozone [26]. It is known that even a small modification in the *IL1RN* gene has the potential to alter inflammatory and immune functions mediated by IL1 binding [18]. 

Several genome-wide linkage studies and genome-wide association studies (GWAS) suggest that the human chromosome region 2q122–q14 containing the IL1 cluster harbors candidate genes for asthma and other inflammatory diseases [10,29,30,31,32,33,34,35,36,37,38]. Associations between single nucleotide polymorphisms (SNPs) in *IL1RN* and asthma were first identified by Gohlke et al. in parent-affected child trios from Germany and Sweden and replicated using an independent cohort of trios from Italy [39]. The design of the study was very robust in avoiding population stratification and the data analyses were careful and thorough. Thus the reported association finding was highly trustworthy, supported by animal and mechanistic studies [24,40]. Moreover, the association between *IL1RN* variants and asthma was reaffirmed independently by Pattaro et al. in an adult German population-based sample [41]. The studied SNPs in *IL1RN* are in strong linkage disequilibrium (LD) [39]. Importantly, in the adult population, carriers of a common haplotype of *IL1RN* involving the common allele “A” in SNP rs2234678 have a high prevalence of doctor-diagnosed asthma (odds ratio OR = 3.12, p = 0.007) [41], in line with findings in pediatric cohorts studied by Gohlke et al. [39] These reported findings suggest that the minor allele “G” and rare genotype GG at SNP rs2234678 would be protective for asthma.

In contrast to these studies, Ramadas et al. reported that the rare genotype GG at rs2234678 carried a significant risk for asthma in a group of children with exposure to maternal smoking during pregnancy (OR = 4.43, p = 0.004) [14]. Thus, the large ORs in these reported studies diverge in opposite directions with the genotype rs2234678 GG carrying a protective effect for asthma in adults [39,41] but a significant risk for asthma in a subgroup of children with passive exposure to tobacco smoke and maternal smoking during pregnancy [14]. The dataset reported in Ramadas et al. was from a well-designed longitudinal cohort study of children who were evaluated up to 10 years old in the United Kingdom. The reported large OR for early onset asthma is likely reproducible. Consequently, these seemingly inconsistent findings between Ramadas et al. and Pattaro et al. highlight an unaddressed important knowledge gap on the impact of *IL1RN* variants on asthma susceptibility in the context of possible gene-environment interactions (GEI). In addition to potential GEI, there is also a knowledge gap between genotypic and haplotype-based SNP analyses. It is well known that genotype of a tag SNP is directly observable and straightforward for analysis, however, the genotype of a tag SNP is likely in LD with a functional variant but itself is unlikely to have direct biological functions. While long haplotypes are likely harboring functional polymorphisms, unfortunately, the linkage phase between SNPs is often ambiguous and the haplotypes cannot be directly observed in typical association studies. It is thus of some importance to infer haplotypes using suitable statistical techniques and reconcile the haplotype findings with more direct genotypic SNP analysis. 

Environmental tobacco smoke (ETS) or passive exposure to tobacco smoke is a well-known risk factor for the development of asthma [42,43,44] and exacerbates asthma symptoms in children and adults [44,45,46,47]. ETS is also an important environmental exposure when assessing genetic susceptibility of asthma [6,12,13,14,15,48]. It is possible that the lungs and immune system of children are more susceptible to injuries due to the local environment including childhood ETS because they are not fully developed. Maternal smoking as studied in Ramadas et al. may attenuate innate immune function in the neonatal period [49], making this a vulnerable time for gene and environment interactions. Epigenetic changes that occur in early life may also contribute to genetic differences in pediatric asthma susceptibility to ETS [50]. However, the GEI or differential association of childhood ETS exposure and risk for asthma or early onset asthma within various *IL1RN* genotype groups has not been reported. The New York University/Bellevue Asthma Registry (NYUBAR) was designed for case-control studies using an admixed urban adult population with a focus on severe asthma [51,52] and collected information on childhood ETS exposure and age at asthma onset. Thus, our main goal was to identify the gene–environment interaction of childhood ETS exposure with *IL1RN* variants and asthma risk and evaluate the impact of GEI on the risk of asthma and early onset asthma to bridge the knowledge gap in understanding the seemingly diverging effects of some rare *IL1RN* genotypes on asthma susceptibility, in contrast to haplotype-based findings in existing cohort studies. A final issue to be addressed is that Pattaro et al. considered asthma in an adult cohort while Ramadas et al. studied pediatric or early-onset asthma for subjects with childhood ETS. At NYUBAR, we have adult asthmatics with information on both childhood ETS exposure and age of onset for asthma, thus we also aimed to bridge the findings on significant protective effect of GG in SNP 2234678 on adult asthma as well as high risk effect on early onset asthma in the presence of childhood ETS exposure.

## 2. Methods 

### 2.1. Study Population and Phenotype Ascertainment

Asthmatics and healthy controls were identified from the New York University Bellevue Asthma Registry (NYUBAR) in New York City [52,53]. The registry is approved by the Institutional Review Board of the New York University School of Medicine and all cases and controls signed informed consent. 

Cases were referred to the registry by the Bellevue Hospital Center Asthma Clinic and local clinics. Controls (unrelated to cases) were referred by asthma cases and by enlisting individuals directly from the community and other programs within Bellevue Hospital Center. Subjects were excluded if they were less than 18 years old, were current smokers or had a history of >10 pack-year tobacco use, had unstable cardiac disease, lung disease other than asthma, or neuromuscular disease.

Questions about ETS exposure were added to questionnaires after the NYUBAR had been initiated and thus the ETS exposure information was not available for a subset of early enrolled subjects. The childhood ETS exposure question asked in the questionnaires is a Yes/No question: ‘‘When you were a child, did you regularly live with someone who smoked?” An answer “Yes” was considered as evidence of early childhood ETS exposure. 

To assemble the case-control study, 259 asthma cases and 182 genetically unrelated healthy controls with childhood ETS exposure information were selected. Participants were ascertained with a diagnosis of asthma as previously described [52] and most had persistent asthma. Measurements of total serum IgE (total IgE) and allergen-specific IgE for allergens significant for the Northeastern United States were performed in a commercial laboratory (Pharmacia ImmunoCAP assay, Quest Diagnostics, Teterboro, NJ). An allergen-specific IgE level > 0.35 kilo-international units (kIU)/L was considered positive. Pre- and post-bronchodilator spirometry was performed according to American Thoracic Society guidelines [54] and normal values were obtained from Hankinson et al. [55]. Individuals were on a stable dose of medications for one month prior to study, but medications were withheld for six hours prior to testing. “Early onset asthma” was defined as a doctor diagnosis of asthma before age 10, to enable comparison with published literature on *IL1RN* [14].

### 2.2. Candidate SNP Selection and Genotyping

Selection of candidate SNPs for genotyping in *IL1RN* was based on the concept of tagging SNPs and haplotypes [56] and the results from three independent asthma studies on *IL1RN* polymorphisms [14,39,41]. Since SNPs in *IL1RN* have been shown to be in strong LD and six tag SNPs were found sufficient for tagging all inferred haplotypes with prevalence above 1% [39] these haplotype tagging SNPs were used to provide a basis for selection of candidate SNPs for a rapid candidate–gene study for *IL1RN*. We replaced the first tagging SNP rs315934 by the SNP rs2234678 since SNP 2234678 is the only SNP discussed in all three published *IL1RN* asthma studies [14,39,41]. The selected six SNPs (i.e., rs2234678, rs392503, rs1794067, rs598859, rs973635, rs440286) can potentially produce long haplotypes covering seven out of the eight exons of the *IL1RN* gene. 

Genotyping of the candidate SNPs was performed on an Illumina BeadStation 500G Golden Gate custom panel (Illumina, Inc., San Diego, US) using unamplified DNA extracted from blood at the Robert S. Boas Center for Genomics and Human Genetics. Genotyping reproducibility was verified with duplicates. The six tag SNPs in *IL1RN* were successfully genotyped with call rate greater than 99%.

### 2.3. Statistical Analyses

Data from categorical variables were summarized using counts and percentages, and compared using chi-square test or Fisher’s exact test. Continuous variables were summarized using mean and standard deviations (SD), and compared using the Mann–Whitney test (MWT). The SNPs were tested for Hardy–Weinberg equilibrium (HWE). 

**Figure 1:**
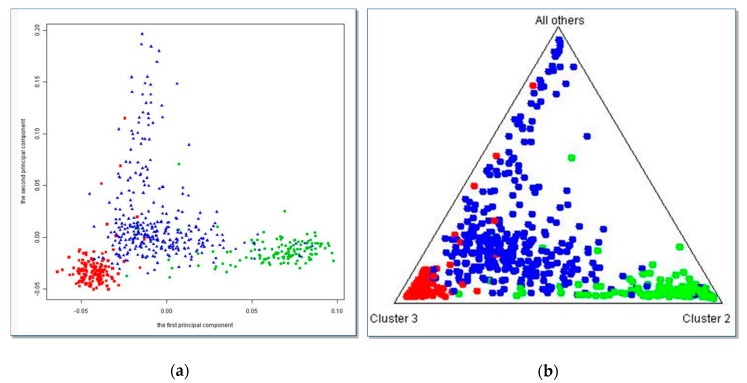
(**a**) Scatterplot of the first and second principal components from the PCA method using 213 ancestral informative markers (AIMs); (**b**) Scatterplot from the STRUCTURE method using 213 AIMs. Red dots indicate self-reported non-Hispanic white; blue dots indicate self-reported non-Hispanic black; green dots indicate self-reported Hispanic. The two methods provided good and similar separation results for the self-reported race/ethnicity groups.

To adjust for population stratification, genotype data on 213 ancestral informative markers (AIMs) were selected for differentiating continental origins most likely to be represented in our cases and controls and to maximize allele frequency differences between ethnic groups including diverse Hispanic ancestry [52,57,58]. For dimension reduction, the principal component analysis (PCA) method [59] or the Bayesian STRUCTURE method [60] were implemented using these AIMs to adjust for population stratification. Principal components were calculated without assigning any particular membership for each subject. The first two principal component scores showed good separation between the self-reported Hispanics, non-Hispanic white group and the non-Hispanic black group (Figure 1a) and the first five components accounting for over 80% of variability of the ancestry markers. Thus these five principal components can be used as covariates to adjust for ancestry admixture in our cohort [52]. The STRUCTURE analysis estimates the posterior probability that each subject belongs to each underlying population at an individual level using a Bayesian approach. Figure 1b shows that the STRUCTURE method had similar results to the PCA method. Thus, in subsequent analyses, the first five principal component scores (PCs) of the AIMs were included as ancestry informative covariates in logistic regression models to adjust for population stratification. 

Separate multiple logistic regression models were fit for susceptibility to asthma and early onset asthma. In particular, tests for SNP genotype (rare versus common) association with asthma and early onset asthma were performed. The logistic regression was used to adjust for age, gender, BMI, education, and ancestry covariates. Haplotypes were reconstructed using the EM algorithm [61] and the haplotype-specific associations were assessed using the score test approach based on the generalized linear model [62,63] using the R package haplo.stats. These results were also validated using the software package for haplotype estimation (PHASE v2) [64,65]. Gene–environment interactions (GEI) for the risk of asthma and early onset asthma were evaluated via stratification on childhood ETS exposure (with/without) and on *IL1RN* SNP genotypes (rare/common), respectively. The significance of GEI was also assessed using conventional method via logistic regressions, adjusted for age, gender, BMI, education and ancestry covariates.

## 3. Results

### 3.1. Population Characteristics

The baseline demographic and clinical characteristics of the case-control study population (N = 259 cases, 182 controls) and the subpopulations stratified by childhood ETS exposure (N = 230 with childhood ETS; N = 211 without childhood ETS) are summarized in Table 1. A majority of the cases and controls were women, and many self-reported as Hispanic. On average, cases were older and had higher BMI than controls (p < 0.002). There were also statistically significant differences between cases and controls in education and income (p < 0.001). Consequently, we adjusted for age, gender, BMI, education, and ancestral distributions in our association analyses. Due to the correlation between income and education, only findings with adjustment for education and other covariates were reported.

Allelic and genotypic frequencies of the six selected tag SNPs of *IL1RN* in the study population are reported in Table 2. Two of the six tag SNPs, rs598859 and rs973635, were not significantly associated with asthma in the original *IL1RN* study reported by Gohlke et al. [39], and thus were not included for subsequent association analyses except haplotype analysis. Moreover, rs598859 was also excluded for haplotype analysis due to violation of the Hardy–Weinberg equilibrium assumption, which is an assumption used in typical haplotype phasing algorithms. For an easy exposition, we focus on the first three SNPs in Table 2 in subsequent SNP genotype association analyses.

### 3.2. Association of IL1RN Genotypes with Asthma and Early Onset Asthma Stratified by Childhood ETS

We first focused on conducting stratified association analyses that would facilitate comparison with findings of both Pattaro et al. [41] on adult asthma in the general population and Ramadas et al. on early onset asthma in the presence of childhood ETS exposure. Ramadas et al. studied three tag SNPs of *IL1RN* with SNP rs2234678 as representative SNP due to a high level of LD among the tag SNPs. For easy comparison, we similarly focused on the first three tag SNPs in Table 2 with SNP rs2234678 as representative SNP. 

To avoid potential confounding of ETS exposure and GEI on assessing the protective effect of GG as reported in Pattaro et al. [41], we focus on the subjects without childhood ETS exposures. Thus, to compare with findings of Pattaro et al. [41] that the rare genotype, GG at rs2234678, would be protective for asthma in general population, genotypic association analysis using asthma as the phenotype among participants without childhood ETS exposure (N = 211) was conducted and reported in Table 3. The rare genotypes were clearly significantly associated with decreased asthma risk in the group without childhood ETS (e.g., OR = 0.215, p = 0.021 for SNP rs2234678). Thus, in the group without childhood ETS exposure, our data indicated that GG in SNP rs2234678 and rare genotypes in other tag SNPs of *IL1RN* were associated with significantly reduced risk (p < 0.05) of asthma susceptibility (or protective effect) relative to the common genotypes. This result was indeed consistent with the findings of Pattaro et al. [41]. However, this was in contrast to those with childhood ETS exposure, shown in our data in Table 3, where GG in SNP rs2234678 and rare genotypes in other tag SNPs of *IL1RN* were associated with a significantly elevated risk (p < 0.05) for early onset asthma.

Environmental tobacco smoke (ETS) is a known risk factor for the development of asthma [42,43,44,66] and exacerbates asthma symptoms in children and adults [44,45,46,47]. ETS is also an important environmental exposure when assessing genetic susceptibility of asthma [6,12,13,14,15,48]. Since Ramadas et al. showed that the rare genotype GG at SNP rs2234678 carried a significant risk for asthma in children less than 10 years old with exposure to maternal smoking during pregnancy [14], we examined the interaction between *IL1RN* genotypes and childhood ETS exposure on the risk of early onset asthma in the NYUBAR. Early-onset asthma is defined as the age of onset less than 10 years old to facilitate comparison with findings reported in Ramadas et al. In particular, genotypic analysis stratified by childhood ETS exposure and risk for early onset asthma is shown in the right column of Table 3. Indeed, among the children with childhood ETS exposure (N = 230), carriers of the *IL1RN* rare homozygous genotypes had a significantly higher risk of developing early onset asthma compared to the carriers of other more common genotypes. For example, among those with childhood ETS exposure, a higher risk of early onset asthma was noted for carriers of rs2234678 GG versus GA/AA (OR = 4.467, p = 0.021). The OR was similar to the earlier finding of Ramadas et al. [14] obtained from repeated measures for children with ETS exposure and maternal smoking during pregnancy (OR = 4.43, p = 0.004). In our data, the OR remained high and similar and significant even after selecting any age between 8 and 12 years as the cutoff age for the definition of early onset asthma (in Appendix A Table A1) suggesting that the results were stable with respect to the selection of the cutoff age. 

### 3.3. Differential Impact of Childhood ETS for Early Onset Asthma within IL1RN Genotype Groups

The differential association of childhood ETS exposure with early onset asthma within various *IL1RN* genotype groups has not been reported in the literature. For association study of the whole case-control population (N = 441), when ignoring information on SNP genotypes of *IL1RN*, childhood ETS exposure was associated with an elevated risk for early onset asthma in multivariate logistic models adjusted for age, BMI, gender, education, and ancestral information (OR = 1.683, p = 0.034). This is in line with the findings in the literature.

We also stratified on genotypes of *IL1RN* (rare versus common) and examined the impact of childhood ETS exposure on the risk of early onset asthma and reported results in Table 4. Childhood ETS exposure significantly increased the risk (p < 0.05) of early onset asthma among subjects with rare genotypes of all the three tagging SNPs of the *IL1RN* with the observed ORs in the range of 8.5 to 9.1, as reported in Table 4. These ORs appeared notably higher than those with more common genotype where the impact of childhood ETS on early onset asthma was not significant (p > 0.05). Thus, there is a clear differential impact of childhood ETS on the risk of early onset asthma between the rare and common genotype groups, indicating GEI between the SNP genotypes and childhood ETS exposures. 

Note that, the conventional method to show the interaction between *IL1RN* SNP genotypes (rare/common) and childhood ETS exposure (Y/N) is to use multivariable logistic regression with both childhood ETS exposure status and SNP genotypes as predictors, and also including their interaction terms with adjustment for age, gender, BMI, education, and AIMs. We conducted this conventional logistic regression analysis and the interaction terms between ETS exposure status and SNP genotypes are all significant for all the three SNPs reported in Table 3 and Table 4 for early onset asthma (rs2234678: p = 0.05; rs392503: p = 0.04; rs1794067: p = 0.03). Thus, the GEI between *IL1RN* tag SNPs and childhood ETS exposure for early-onset asthma is indeed significant in this study.

Similar to the association analysis of early-onset asthma, it is important to account for the potential effect of GEI in genotypic association SNP analysis for asthma in our data to avoid false negatives. As an example, the genotypic association analysis results of SNP rs2234678 for asthma are presented in Table 5 with N = 441. Notably, without adjusting for the GEI between the rare GG at SNP rs2234678 and childhood ETS, the protective effects of the rare SNP genotype GG at SNP rs2234678 for asthma relative to their common genotypes was not significant with or without adjustment for ETS. Thus the association between GG and asthma would not be detectable in typical genotypic analysis when GEI is ignored. On the other hand, when the GEI term (GG*ETS) and childhood ETS were included in the logistic regression with adjustment of age, gender, BMI, education, AIMs, and other covariates, the protective effect of GG is statistically significant at the 5% level. Thus, ignoring potential GEI in SNP association analysis can cause serious loss of statistical power and false non-discoveries. 

### 3.4. GEI of IL1RN SNP Haplotypes and Childhood ETS Exposure for Early Onset Asthma Susceptibility

To reconcile the genotypic analysis results from UK pediatric cohort and NYUBAR with haplotype analysis findings in the German asthma cohorts [39,41,67], we also investigated the impact of the haplotypes of the tag SNPs using the generalized linear regression method for haplotypes [62,63]. The long haplotype GGAGA (frequency = 0.211) in rs2234678-rs392503-rs1794067-rs973635-rs440286 had a protective effect (OR = 0.62, p = 0.01) for asthma relative to the common haplotype AAGGC (frequency = 0.475) in the study population (N = 441). These results were consistent with the previously published findings in German populations [39,41]. Thus these results from nonstratified analyses using our admixed cohort supported the previously reported finding in the German population [39,41] that several candidate *IL1RN* SNPs and haplotypes have association with asthma susceptibility. Also, haplotype analysis indicates differential impact of childhood ETS on different haplotypes as reported in Table 6.

As pointed out by Gohlke et al., the studied SNPs in *IL1RN* are in strong linkage disequilibrium (LD) [39]. Indeed, the haplotype frequency of GGAGA (frequency = 0.211) in the study population is approximately equal to the observed allelic frequency of the minor allele “G” in SNP rs2234678, and “G” in SNP rs392503, and “A” in SNP rs1794067, etc., as reported in Table 2. Therefore, the people with rare homozygous SNP genotypes, e.g., GG in SNP rs2234678, most likely have two protective GGAGA haplotypes for asthma, thus they have a low risk for asthma and early onset asthma at least when not exposed to childhood ETS. In particular, from Table 6, when restricted to the subgroup of subjects without childhood ETS exposure (N = 211), the haplotype GGAGA also has a protective effect for asthma (OR = 0.54, p = 0.03), and (OR = 0.89, p = 0.74, not shown in Table 6) for early onset asthma relative to the common haplotype AAGGC. However, from Table 6, among the subjects with childhood ETS exposure (N = 230), the haplotype GGAGA loses its protective effect and seemingly carries a higher risk for early onset asthma (OR = 1.42, p = 0.23) than the reference haplotype AAGGC, which itself carries the risk for asthma. Consequently, it is possible that the subjects with the rare homozygous SNP genotype GG in SNP rs2234678 have two risk GGAGA haplotypes, thus they have decreased risk for asthma or early onset asthma without exposure to ETS but have increased risk for early onset asthma when exposed to childhood ETS. This haplotype analysis further bridges the knowledge gap in the literature between the seemingly contradicting genotypic-based association results from the UK longitudinal cohort study and the haplotype-based association results from Germany cohort-based studies, and also indicates the plausibility of the observed GEI for rare genotypes of the tag SNPs in *IL1RN*. Of course, when stratified by childhood ETS exposure status, the sample sizes are much smaller in each subgroup, so the statistical significance of the findings would be sacrificed. Importantly, the haplotype analyses reported in Table 6 also indicate that subjects with haplotype AAGAC (frequency = 0.223) or haplotype AAAGC (frequency = 0.057) also have protective effect for asthma among those without childhood ETS exposure (OR < 1), but have increased risk for early onset asthma (OR > 1) among subjects exposed to childhood ETS. Thus, while it is easier to identify GEI using subjects with rare homozygous tag SNP genotypes/haplotypes, from Table 6, the childhood ETS exposure clearly has differential negative impact on subjects far beyond that seen in the small proportion with rare homozygous tag SNP genotypes/haplotypes. Thus the GEI and negative impact of childhood ETS were not merely affecting small negligible subpopulations, and they can potentially have significant adverse health impacts in general populations.

## 4. Discussion

Genotypic/haplotype analyses of the tag SNPs in *IL1RN* and asthma susceptibility for the NYUBAR population as a whole resulted in findings that were in accordance with previous publications using different analyses and independent cohorts [39,41,67]. Our data support the evidence of the protective effects of some SNP alleles and haplotypes for asthma previously identified in other cohorts [39,41,67]. Importantly, our analyses indicated that these protective effects of these tag SNP polymorphisms were significant only within the groups without childhood ETS exposure and in fact, these polymorphisms became risk variants for early onset asthma in the subgroup exposed to childhood ETS. The latter finding is consistent with the findings of Ramadas et al. [14] using a cohort from the United Kingdom. Our data, therefore, suggest that childhood ETS exposure modifies the *IL1RN* haplotype/genotype-dependent risk for asthma susceptibility and bridge a knowledge gap and explain previous discordant study results on GEI between variants of *IL1RN* and ETS exposure in asthma studies. 

Numerous studies suggest that the asthma risk of genetic variants might be modified in the presence of environmental factors such as ETS exposure [12,13,14,68]. Linkage studies indicate that multiple chromosomal regions, including the 2q region harboring the IL1 cluster, contain risk variants that make carriers of these variants who are exposed to ETS more susceptible to asthma [12,13]. When stratified by genotypes of *IL1RN* and focused on subjects with rare homozygous genotypes of *IL1RN*, we found that childhood ETS exposure elevated the risk for early onset asthma. When stratified by childhood ETS exposure, those with childhood ETS and the rare homozygous genotype of *IL1RN* had a higher risk for early onset asthma compared to those with common genotypes in multivariate analysis. In particular, the finding of a higher risk of early onset asthma for carriers of rs2234678 GG versus rs2234678 GA/AA (OR = 4.467, p = 0.021) is consistent with the increased risk in children with maternal ETS exposure (OR = 4.43, p = 0.004) reported by Ramadas et al. [14] In addition, the finding of a much higher risk of early onset asthma for carriers of rs2234678 GG in the childhood ETS exposed group is in striking contrast to our data in those without childhood ETS, in which the rare genotype GG at rs2234678 was significantly associated with a smaller risk of asthma susceptibility (OR = 0.215, p = 0.021). These results are in support of published results of Pattaro et al. [41] who studied an adult German population-based sample and reported that carriers of a common haplotype of *IL1RN* involving the common allele “A” in SNP rs2234678 have a high prevalence of doctor-diagnosed asthma. Thus the finding of Pattaro et al. ^41^ would imply GG in SNP rs2234678 would have a protective effect as indicated in Table 3 in the group without ETS exposure for asthma.

The finding in the stratified analysis of *IL1RN* variants with odds ratios in opposite directions reinforces the importance of accounting for both environmental exposures and GEI in risk analyses for asthma. The findings suggest that in such situations, association analyses that ignore environmental exposures and GEI can potentially lead to false negatives or overly conservative p-values. This is illustrated by the genotypic association analysis of SNP rs2234678 in Table 3 and Table 5. The sample size for the group without ETS exposure (N = 211) is much smaller than the overall sample size N = 441, however, the p-value is significant among those without childhood ETS (p = 0.021, Table 3), but is not significant in the overall group (N = 441) for SNP rs2234678 (p = 0.07, Table 5). Similarly, GWAS without considering GEI can also miss allelic associations. In addition, analyses that do not adjust for environmental exposure can easily cause confusion and paradoxical findings. For example, from Table 5, the nonstratified analysis (N = 441) found a nonsignificant protective effect (OR = 0.459, p = 0.07) for rs2234678 GG, which in fact merely reflected the significant effect among subjects without ETS exposure (OR = 0.215, p = 0.021) as reported in Table 3. On the other hand, rs2234678 GG was found to be a nonsignificant risk genotype (OR = 1.788, p = 0.203) for early onset asthma in nonstratified analysis, which merely reflected the significant effect among subjects with childhood ETS exposure (OR = 4.467, p = 0.021, Table 3). Furthermore, in the nonstratified analysis for the overall group (N = 441), using SNP rs2234678 as an example, the OR of the rare genotype GG was less than one for susceptibility to asthma (Table 5, neither adjusting for ETS nor GEI) whereas it was larger than one for susceptibility to early onset asthma (data not shown). Similar results hold for other SNPs. This paradox illustrates the potential confusion which can be caused by failing to account for important environmental risk factors such as ETS.

The divergent findings of an elevated risk for the interaction between *IL1RN* genotypes and childhood ETS for early onset asthma but not for asthma in the total population have precedent in the literature on childhood predisposition to asthma. The linkage associations between the “Th2” cytokine gene cluster and asthma susceptibility have been suggested to be more evident in children exposed to tobacco smoke [12,13]. Divergent effects of exposures have been shown for CD14 and pets with ETS exposure [69]. Although we have not examined potential explanations for the age change in susceptibility, the possibility exists that the lungs and immune system of children are more susceptible to injuries due to the environment including childhood ETS because they are not fully developed. Maternal smoking may attenuate innate immune function in the neonatal period [49], making this a vulnerable time for gene and environment interactions. Epigenetic changes that occur in early life may also contribute to genetic differences in susceptibility to ETS [50]. Recent studies on CD14, in which divergent effects have also been shown, suggest that the presence of pets and ETS limit the increase in CD14 methylation that occurs before age 10 years and this has been suggested as a partial explanation for the diverging CD14 allele associations with allergic diseases detected in different environments [70].

Studies on GEI can have significant implications for public health. Childhood ETS exposure was a risk factor for early onset asthma for our study population regardless of genotypes even after adjustment for many covariates. Moreover, childhood ETS exposure led to significant elevation of risk for early onset asthma for the subpopulation who carried rare genotypes of the tag SNPs of *IL1RN* including the GG genotype in SNP rs2234678. Childhood ETS exposure was about 52% in our study cohort, consistent with current estimates suggesting that 55% of children have detectable levels of serum cotinine [71]. Given that childhood ETS exposure is the most common but preventable childhood environmental hazard, our findings reinforce the impact of childhood ETS exposure on early onset asthma. 

The use of an admixed population can complicate genetic analyses. We accounted for population admixture with the use of ancestral markers and after adjustment for population admixture, our findings were consistent with previous studies that did not have the potential biases of population stratification when using the transmission disequilibrium/test (TDT) with parents-affected child trios. After adjusting for age, BMI, gender, and ancestral information, many rare haplotypes of *IL1RN* tagging SNPs rs2234678-rs392503-rs1794067-rs973635-rs440286 (e.g., GGAGA) had protective effect for asthma relative to the more common haplotype AAGGC in either the overall population or subjects without childhood ETS exposure. The haplotype AAGGC is more common than other haplotypes, but its frequency is less than 50%. This finding was consistent with previously published reports [39], suggesting that the finding was robust across different ancestries. Moreover, we previously replicated genetic and asthma phenotype studies with this population [52,53], supporting the robustness of this population for asthma analyses. 

There are several potential limitations to this study. We recruited an adult population and did not have extra information to confirm childhood ETS exposure (i.e., cotinine measurements), thus the responses about the exposure to childhood ETS may be subject to recall bias. However, childhood ETS exposure is about 52% in our study cohort, which is consistent with current estimates suggesting that 55% of children have detectable levels of serum cotinine [71]. We do not have information about in utero tobacco exposures. We suspect some of those with childhood ETS exposure might have maternal smoking during pregnancy as studied in Ramadas et al. [14], but we do not have that information. A similar potential for recall bias exists for age of onset of disease. We used physician diagnosis to determine age of onset of asthma, however, asthma symptoms may pre-exist age of diagnosis. These biases would not be expected to differ systematically between the comparison groups. We selected a cut point of age 10 as early onset asthma to be comparable with previous studies of *IL1RN* [14]. However, the use of any cutoff point between ages 8 and 12 years did not affect the association results of our analysis (Table A1), suggesting that the analysis method we employed was robust against potential recalling errors on age of onset. Finally, after stratification for ETS, our rare genotype groups became quite small. It is possible that epigenetic mechanisms are involved in the gene–environment interaction in the context of childhood ETS exposure, unfortunately, we do not have data on epigenetic mechanisms for the study. Our ability to identify a strong OR in the subpopulations, suggested that the effect of the association was quite strong, a finding that is consistent with the fact that both Pattero et al. and Ramadas et al. found strong effect sizes (large ORs). Nevertheless, we recognize the desirability for additional replication in even larger samples for the GEI finding.

## 5. Conclusions

Both genetic and environmental factors and their interactions contribute to the highly complex etiology of asthma. Environmental factors include passive tobacco exposure, which may disrupt normal gene functions including those in the innate immune pathway. The study of gene–environment interactions may lead to a better understanding of the pathological processes and biological mechanisms that contribute to the development of complex diseases. We now report population level evidence that a common environmental exposure can disrupt the function of protective polymorphisms of a specific immune/anti-inflammation gene and turn it into significant risk polymorphisms for the early onset of a complex disease among exposed children. Given that childhood ETS exposure is preventable and still common [71], these findings have public health implications and reinforce the need to further reduce childhood ETS exposure. 

## Figures and Tables

**Table 1:** Characteristics of study population.

	Total			With ETS			Without ETS		
Characteristic	Case	Control	p-Value *	Case	Control	p-Value *	Case	Control	p-Value *
	(N = 259)	(N = 182)		(N = 144)	(N = 86)		(N = 115)	(N = 96)	
Gender, N (%)			0.2			0.98			0.11
Female	183 (70.7)	118 (64.8)		97 (67.4)	57 (66.3)		86 (74.8)	61 (63.5)	
Male	76 (29.3)	64 (35.2)		47 (32.6)	29 (33.7)		29 (25.2)	35 (36.5)	
Age, Mean (SD)	38.7 (14.0)	36.0 (11.6)	0.11	40.4 (14.1)	37.2 (11.7)	0.14	36.5 (13.6)	34.9 (11.4)	0.64
BMI, Mean (SD)	29.7 (7.4)	27.2 (5.6)	< 0.001	30.5 (7.8)	27.4 (5.6)	< 0.001	28.8 (6.7)	27.0 (5.6)	0.05
Income, N (%)			< 0.001			< 0.002			0.01
<15,000 USD per year	108 (41.7)	40 (22.0)		66 (45.8)	22 (25.6)		42 (36.5)	18 (18.8)	
15,000 to 49,999 USD	71 (27.4)	68 (37.4)		32 (22.2)	28 (32.6)		39 (33.9)	40 (41.7)	
>= 50,000 USD	46 (17.8)	57 (31.3)		29 (20.1)	31 (36.0)		17 (14.8)	26 (27.1)	
NA	34 (13.1)	17 (9.3)		17 (11.8)	5 (5.8)		17 (14.8)	12 (12.5)	
Education, Mean (SD)	12.6 (3.8)	14.3 (3.5)	< 0.001	12.8 (3.8)	14.2 (3.8)	0.003	12.5 (3.7)	14.3 (3.3)	< 0.001
Race/Ethnicity, N (%)			0.08			0.89			0.02
Hispanic	151 (58.3)	87 (47.8)		77 (53.5)	44 (51.2)		74 (64.3)	43 (44.8)	
Non-Hispanic Black	51(19.7)	48 (26.4)		28 (19.4)	19 (22.1)		23 (20.0)	29 (30.2)	
Non-Hispanic White	57 (22.0)	47 (25.8)		39 (27.1)	23 (26.7)		18 (15.7)	24 (25.0)	
Smoking Status, N (%)			0.2			0.61			0.24
Ex-smokers	71 (27.4)	40 (22.0)		43 (29.9)	23 (26.7)		28 (24.3)	17 (17.1)	
Never-smokers	188 (72.6)	142 (78.0)		101 (70.1)	63 (73.3)		87 (75.7)	79 (82.3)	
Spirometry									
FEV_1_, % predicted	79.2 (16.8)	91.4 (11.8)	< 0.001	78.7 (15.9)	90.7 (12.1)	< 0.001	79.8 (18.0)	92.0 (11.6)	< 0.001
FVC, % predicted	86.7 (15.4)	92.7 (12.5)	< 0.001	85.8 (14.6)	92.3 (13.3)	0.001	87.9 (16.3)	93.0 (11.7)	0.02
FEV_1_/FVC	74.7 (9.2)	81.5 (5.2)	< 0.001	74.6 (9.3)	80.8 (4.9)	< 0.001	74.9 (9.2)	82.1 (5.5)	< 0.001
Total IgE, geometric mean	114.4	52.5	< 0.001	106.3	50.7	0.001	125.5	54.1	< 0.001
Atopic status, N (%)			< 0.001			0.02			< 0.001
No	60 (23.2)	78 (42.9)		37 (25.7)	36 (41.9)		23 (20.0)	42 (43.8)	
Yes	199 (76.8)	104 (57.1)		107 (74.3)	50 (58.1)		92 (80.0)	54 (56.2)	

* Chi-square test for categorical variables and the Mann–Whitney–Wilcoxon test for continuous variables. FEV_1_: forced expiratory volume in one second; FVC: forced vital capacity; IgE: Immunoglobulin E.

**Table 2:** Tag *IL1RN* SNPs allele and genotype frequencies in New York University Bellevue Asthma Registry (NYUBAR).

SNP	Chromosomal Location	Alleles	Allele Frequency	Genotype	Genotype Frequency	HWE * Test p-Value
rs2234678	113875565	A/G	0.78/0.22	AA/AG/GG	0.61/0.33/0.06	0.17
rs392503	113884195	A/G	0.77/0.23	AA/AG/GG	0.60/0.33/0.07	0.18
rs1794067	113886384	G/A	0.71/0.29	GG/GA/AA	0.52/0.40/0.09	0.64
rs598859	113888134	C/A	0.82/0.18	CC/CA/AA	0.64/0.36/0.00	< 0.001
rs973635	113889134	G/A	0.77/0.23	GG/GA/AA	0.60/0.33/0.06	0.22
rs440286	113889469	C/A	0.77/0.23	CC/CA/AA	0.61/0.33/0.06	0.28

* HWE: Hardy–Weinberg equilibrium.

**Table 3:** Genotypic association for asthma and early onset asthma stratified by childhood ETS.

SNP	Genotype	Asthma		Early Onset Asthma	
		*without* Childhood ETS (N = 211)		*with* Childhood ETS (N = 230)	
		OR *	p-Value *	OR *	p-Value *
rs2234678	AG&AA	1		1	
	GG	0.215	**0.021**	4.467	**0.021**
rs392503	AA&AG	1		1	
	GG	0.215	**0.021**	4.577	**0.012**
rs1794067	GG&AG	1		1	
	AA	0.218	**0.008**	3.123	**0.032**

* Logistic regression adjusted for age, BMI, gender, education, ancestral informative markers (AIMs) (5PCs). OR: odds ratio. Bold number for p-value means statistically significant at the 5% level.

**Table 4:** Odds ratios of childhood ETS exposure on early onset asthma by SNP genotype subgroups.

Rare Genotype						Common Genotype					
SNP (Genotype)	ETS	Asthma Onset ≤ 10 yrs, N	Asthma Onset > 10 yrs, N	OR	p-value	SNP (Genotype)	ETS	Asthma Onset ≤ 10 yrs, N	Asthma Onset > 10 yrs, N	OR	p-value
rs2234678 (GG)	Yes	7	5	9.1	**0.037**	rs2234678 (AG&AA)	Yes	55	150	1.5	0.123
	No	2	13				No	38	152		
rs392503 (GG)	Yes	8	6	8.7	**0.021**	rs392503 (AA&AG)	Yes	54	150	1.4	0.153
	No	2	13				No	38	152		
rs1794067 (AA)	Yes	9	9	8.5	**0.013**	rs1794067 (GG&AG)	Yes	53	147	1.4	0.149
	No	2	17				No	37	148		

OR: odds ratio. Bold number for p-value means statistically significant at the 5% level.

**Table 5:** Genotypic association of SNP rs2234678 for asthma (N = 441).

	Neither Adjusting for ETS nor GEI		Adjusting for ETS but not GEI		Adjusting for both ETS & GEI	
SNP rs2234678	GG	AG&AA	GG	AG&AA	GG	AG&AA
OR *	0.459	1	0.467	1	0.231	1
95% CI *	(0.19, 1.06)		(0.20, 1.08)		(0.06, 0.76)	
p-value *	0.07		0.08		**0.02**	

* Logistic regression adjusted for age, BMI, gender, education, AIMs (5PCs). GEI: gene–environment interaction. OR: odds ratio. Bold number for p-value means statistically significant at the 5% level.

**Table 6:** Haplotype association for asthma and early onset asthma stratified by childhood ETS.

Haplotype ^+^ (Frequency)	Asthma		Early Onset Asthma	
	*without* Childhood ETS (N = 211)		*with* Childhood ETS (N = 230)	
	OR *	p-Value *	OR *	p-Value *
AAGGC (0.475)	Ref			
GGAGA (0.211)	0.54	**0.03**	1.42	0.23
AAGAC (0.223)	0.94	0.82	1.46	0.21
AAAGC (0.057)	0.93	0.88	1.19	0.73

^+^ Haplotypes of tagging SNPs: rs2234678-rs392503-rs1794067-rs973635-rs440286. * Logistic regression on haplotypes with adjustments of age, BMI, gender, AIMs, etc. OR: odds ratio. Bold number for p-value means statistically significant at the 5% level.

## Appendix A

We have discovered a gene–environment interaction between tag SNPs of the interleukin-1 receptor antagonist (*IL1RN*) gene and childhood ETS exposure for early onset asthma. For easy comparison with published results in Ramadas et al., we used a cut point of age **≤** 10 of asthma diagnosis as early onset (e.g., Table 3). In the Appendix A Table A1, we report the SNP genotypic association of *IL1RN* SNPS with early-onset asthma for different selection of cut points to examine the sensitivity of the association to the selection of the cutoff value used in the logistic regression analysis with adjustment of age, gender, BMI, education, and AIMs. From the table, it is clear that both the odds ratios and the p-values are not affected much by a different selection of the cut point age to define early onset. Therefore, the newly discovered gene–environment interaction in this study is quite robust to the selection of the cutoff point for the definition of early onset asthma.

**Table A1:** SNP genotypic association with early onset asthma using different cutoff for age diagnosed.

SNP	Genotype	Risk of Early Onset Asthma among Subjects with Childhood ETS Exposure (n = 230)									
		Age (in years) Diagnosed									
		≤ 8		≤ 9		≤ 10		≤ 11		≤ 12	
		OR *	p-Value *	OR *	p-Value *	OR *	p-Value *	OR *	p-Value *	OR *	p-Value *
rs2234678	AG&AA	1		1		1		1		1	
	GG	4.941	**0.014**	4.941	**0.014**	4.467	**0.021**	4.395	**0.023**	4.149	**0.031**
rs392503	AA&AG	1		1		1		1		1	
	GG	5.157	**0.007**	5.157	**0.007**	4.577	**0.012**	4.474	**0.013**	4.196	**0.019**
rs1794067	GG&AG	1		1		1		1		1	
	AA	3.506	**0.02**	3.506	**0.02**	3.123	**0.032**	2.978	**0.041**	2.819	**0.05**

* Logistic regression adjusted for age, BMI, gender, education, AIMs (5PCs). OR: odds ratio. Bold number for p-value means statistically significant at the 5% level.
